# Optimizing data processing to improve the reproducibility of single‐subject functional magnetic resonance imaging

**DOI:** 10.1002/brb3.1617

**Published:** 2020-04-19

**Authors:** David A. Soltysik

**Affiliations:** ^1^ Division of Biomedical Physics Office of Science and Engineering Laboratories Center for Devices and Radiological Health Office of Medical Products and Tobacco U.S. Food and Drug Administration Silver Spring MD USA

**Keywords:** functional magnetic resonance imaging, motion correction, reproducibility, spatial smoothing

## Abstract

**Introduction:**

High reproducibility is critical for ensuring the confidence needed to use functional magnetic resonance imaging (fMRI) activation maps for presurgical planning.

**Methods:**

In this study, the comparison of different motion correction methods, spatial smoothing methods, regression methods, and thresholding methods was performed to see whether specific data processing methods can be employed to improve the reproducibility of single‐subject fMRI activation. Three test–retest metrics were used: the percent difference in activation volume (PDAV), the difference in the center of mass (DCM), and the Dice Similarity Coefficient (DSC).

**Results:**

The PDAV was minimized when using little or no spatial smoothing and AMPLE thresholding. The DCM was minimized when using affine motion correction and little or no spatial smoothing. The DSC was improved when using affine motion correction and generous spatial smoothing. However, it is believed that the overlap metric may be unsuitable for testing fMRI reproducibility.

**Conclusion:**

Processing methods to improve fMRI reproducibility were determined. Importantly, the processing methods needed to improve reproducibility were dependent on the fMRI activation metric of interest.

## Graphical abstract

This study reveals data processing methods to improve the reproducibility of single‐subject fMRI. Importantly, the optimum methods depend on the activation metric of interest.

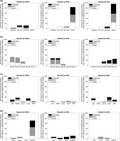

## 1 INTRODUCTION

Currently, the most important clinical application of functional magnetic resonance imaging (fMRI) is presurgical planning to prevent functional deficits (Bookheimer, 2007; Stippich, Blatow, & Garcia, 2015). fMRI data can be used to influence the surgical management of patients by determining the necessity of intraoperative mapping, the necessary extent of brain exposure, and the safest surgical route (Morrison et al., 2016). Achieving fMRI activation maps with high reliability is critical to preserving eloquent motor and language regions during presurgical planning (Nettekoven, Reck, Goldbrunner, Grefkes, & Lucas, 2018).

Despite nearly three decades of research in fMRI, there is little standardization for imaging procedures, data processing, and interpretation of the results (Stippich et al., 2015). Recently, the American Society of Functional Neuroradiology (ASFNR) has published a white paper on recommended paradigms for presurgical language assessment (Black et al., 2017). And the American Academy of Neurology has published a guideline for the use of fMRI in the presurgical evaluation of patients with epilepsy (Szaflarski et al., 2017). However, there remain no guidelines for data processing, which varies widely from site to site. The lack of standardized processing methods suggests that the precision of fMRI activation maps may be variable across sites.

The precision, or reliability, of quantitative imaging can be assessed in test–retest studies (Sullivan et al., 2015). Repeatability is defined as the measurement precision that occurs with near‐identical conditions (“a set of conditions that includes the same measurement procedure, same operators, same measuring system, same operating conditions and same physical location, and replicate measurements on the same or similar experimental units over a short period of time”) (Kessler et al., 2015). Reproducibility is the measurement precision that occurs with “different locations, operators, measuring systems, and replicate measurements on the same or similar objects” (Kessler et al., 2015). If two imaging scans are performed within a matter of minutes or hours, such a test–retest study is considered a repeatability study. If two scans are separated by more time (e.g., days), where more variability is expected, it is considered a reproducibility study.

Measurement accuracy is dependent on both trueness and precision (ISO, 1994). The spatial localization of BOLD fMRI activation is known to be coincident with local field potentials (Logothetis, Pauls, Augath, Trinath, & Oeltermann, 2001). If we assume that the neuronal activation of interest is captured by parenchymal BOLD fMRI and fMRI images are collected with minimal spatial distortion, we can make the assumption of minimal bias or systematic error. With this assumption, improving precision reduces random error, increasing the accuracy of the measurement (Menditto, Patriarca, & Magnusson, 2007). Therefore, any methods that can increase the reproducibility of fMRI activation will improve its accuracy, improving its technical performance.

A review of the fMRI reproducibility literature revealed that many different methods have been used to assess reproducibility with two popular methods being cluster overlap and intraclass correlation coefficients (ICC) (Bennett & Miller, 2010), the former being a cluster‐dependent metric and the latter being a voxel‐dependent metric. General findings suggested that increased amounts of time between scans lower the reliability of results. Sensorimotor tasks were seen to have greater reliability than cognitive tasks. A few studies showed lower reliability in patients with clinical disorders compared to normal controls. Block designs were seen to yield more reliability than event‐related designs. Reliability also varied across studies. Bennett and Miller (2010) concluded that there is no consensus regarding what constitutes an acceptable level of reliability in fMRI.

There are many steps that can be implemented during experimental design and data acquisition to improve the reproducibility of fMRI results. Bennett and Miller (2010, 2013) recommended having a well‐maintained scanner, well‐designed tasks (target vs. nontarget, block designs), training subjects in a mock scanner, and taking advantage of longer scan time when possible. For subjects undergoing multiple sessions, scanning at the same time of day can reduce variability caused by circadian changes in hormone level (Bennett & Miller, 2010). Gorgolewski, Storkey, Bastin, Whittle, and Pernet (2013) discussed how different tasks involve different cognitive strategies and reported that the type of task used could explain 30%–40% of the single‐subject reliability.

The choice of data processing steps also has the potential to impact the reproducibility of fMRI activation. Gorgolewski, Storkey, Bastin, Whittle, Wardlaw, et al. (2013) found that between‐session variance was mostly caused by underlying cognitive processes and motion rather than technical limitations of data processing. However, their study examined the reproducibility of voxelwise time series and voxelwise *t*‐statistics. This represents a rather limited analysis because time‐series variance changes across sessions and cannot be used to examine reproducibility of activation magnitude, as pointed out by Cohen and DuBois (1999). Furthermore, reproducibility metrics that depend on voxelwise values may not be useful for assessing the reproducibility of fMRI metrics important for clinical applications.

In presurgical planning, the determination of eloquent motor and language cortices is important for neurosurgeons in avoiding the loss of motor and language functions in patients. Spatial extent of fMRI activation is often regarded as important as studies have shown that the risk of postoperative deficits decrease when the lesion to activation distance is >10 mm (Håberg, Kvistad, Unsgård, & Haraldseth, 2004) or 20 mm (Voss et al., 2013). Alternatively, Stippich et al. (2015) have argued that weighted centers of mass of activation clusters and identification of anatomical structures are of greater importance, as the spatial extent is highly dependent on the statistical threshold employed. To address the concerns of both spatial extent and localization, test–retest metrics in this study were employed to examine the reproducibility of activation volume and the weighted center of mass.

Early studies showed that motion correction can improve the sensitivity of fMRI (Morgan, Dawant, Li, & Pickens, 2007; Oakes et al., 2005). However, motion correction can also induce artifactual signals. Freire and Mangin (2001) reported that standard motion correction methods could induce spurious activations, increasing the presence of false positives. Grootoonk et al. (2000) showed that nonideal interpolation methods can create residual artifacts during motion correction. Voyvodic (2012) reported that motion correction did not improve reproducibility, but details were not provided. While motion correction is generally applied because of assumed benefits, it remains unclear what effect it has on the reproducibility of fMRI activation and if different motion correction methods may yield different effects on the reproducibility of activation.

Normalization of subject anatomy to an atlas brain can be useful for combining data in a group analysis, but does not provide much benefit for single‐subject studies. Miki et al. (2000) found that spatial normalization does not significantly affect the reproducibility of activation volume or overlap. Özcan, Baumgärtner, Vucurevic, Stoeter, and Treede (2005) found that spatial normalization could lead to activation appearing on the wrong side of the sulcus. They recommended using individual anatomy for improving spatial localization. Swallow, Braver, Snyder, Speer, and Zacks (2003) found that normalization to the Talairach brain produced smaller differences in the centers of mass in a repeatability study, but this is likely due to the fact that the Talairach atlas, which was based on one older woman's brain, is considered to be smaller than an average brain. To illustrate this point, the Talairach atlas brain has been shown to be smaller than the MNI atlas brain, which is based on a population of hundreds of subjects (Lancaster et al., 2007).

Spatial smoothing is a common technique used to increase the image signal‐to‐noise ratio (SNR) based on the inherent spatial correlation of image data, but is known to affect spatial localization (Parrish, Gitelman, LaBar, & Mesulam, 2000). Rombouts, Barkhof, Hoogenraad, Sprenger, and Scheltens (1998) reported that spatial smoothing increased the difference in centers of mass and the overlap index between separate runs, but no statistical tests were performed. Spatial smoothing can also merge activation in anatomically distinct brain regions (Fransson, Merboldt, Petersson, Ingvar, & Frahm, 2002), causing a loss of information on spatial extent and the shape of the activation area (Tabelow, Polzehl, Voss, & Spokoiny, 2006). White et al. (2001) reported that filter sizes above 8 mm resulted in a shift of the center of mass for activation clusters. Furthermore, Geissler et al. (2005) reported an increase in the aberrations of motor activation centers due to spatial smoothing, indicating a significant decrease in localization replicability. More recently, Raemaekers, Du Plessis, Ramsey, Weusten, and Vink (2012) reported that spatial smoothing decreased variability of activation patterns. It remains unclear what amount of spatial smoothing will optimize the reproducibility of fMRI activation.

Regression methods often employ motion parameters as nuisance regressors to remove effects of motion that remain after rigid motion correction. Lund, Nørgaard, Rostrup, Rowe, and Paulson (2005) reported that the use of motion parameters in regression significantly reduced both the intrasubject and the intersubject variance. However, in a functional connectivity study, Van Dijk, Sabuncu, and Buckner (2012) reported that regressing head motion parameters did not change the test–retest reliability of the results. For task‐based fMRI, such regressors were found to reduce the sensitivity of fMRI activation in both block design (Johnstone et al., 2006) and event‐related design studies (Ollinger et al., 2009). In addition, Churchill et al. (2012) reported that the use of motion parameters as nuisance regressors reduced repeatability of fMRI activation. Stevens, D’Arcy, Stroink, Clarke, and Beyea (2013) reported a single case where the use of motion regressors improved reproducibility, but reported reduced sensitivity in subjects where motion was not an issue. It remains unclear if any regression methods used in fMRI analysis can consistently yield an improvement in the reproducibility of fMRI activation.

Statistical threshold can potentially affect the reproducibility of fMRI activation. Several studies (Duncan, Pattamadilok, Knierim, & Devlin, 2009; Fernandez et al., 2003; Nettekoven et al., 2018) reported that lower thresholds yielded higher overlap between fMRI runs for language tasks. Similarly, Stevens et al. (2013) reported that test–retest overlap increased as the threshold decreased for a finger‐tapping task. However, Rutten, Ramsey, Van Rijen, and Van Veelen (2002) reported that more stringent statistical thresholds increased the lateralization index across four different language tasks, decreased the overlap for antonym generation and picture naming, but did not affect the overlap for verb generation or the combined task analysis. Similarly, Fesl et al. (2008) reported that stringent thresholds were necessary to ensure reliable centers of mass. Rombouts et al. (1998) reported that changing the correlation threshold changed the overlap index with a maximum appearing for thresholds somewhat below the Bonferroni threshold. Soltysik et al. (2011) reported that increasing thresholds led to a decrease in repeatability for activation volume and an increase in repeatability for average percent signal change. It is uncertain if a particular, fixed thresholding method can improve the reproducibility of fMRI activation.

Region of interest (ROI) selection has been shown to affect the repeatability of the center of mass. Agarwal et al. (2018) found that the center of mass variability was lowest in Broca's area, slightly higher in Wernicke's area, and the highest when examining the receptive language area.

Although there have been many studies examining the reproducibility of fMRI and the effect of different data processing methods on fMRI activation, no systematic study comparing the effect of many different processing methods on the reproducibility of fMRI activation metrics has been performed. It would be beneficial to know if any particular data processing methods can improve the reproducibility of fMRI activation metrics. For the specific application of presurgical planning, it is of great interest to find methods that can improve the reproducibility of activation volume, the center of mass, and activation overlap.

In this study, a systematic attempt was made to analyze a test–retest fMRI data set, analyzing every possible combination of four different stages of data processing. These stages included motion correction, spatial smoothing, regression, and thresholding. Four to five different methods for each stage were used to compare different approaches. The goal was to compare a limited number of popular fMRI processing methods to see whether particular methods could optimize reproducibility of the three aforementioned fMRI activation metrics. The results of this study can be used to modify data processing pipelines to improve the reproducibility of activation metrics in single‐subject fMRI scans.

This study was performed as part of an effort by the Quantitative Imaging Biomarkers Alliance (QIBA) sponsored by the Radiological Society of North America (RSNA) to help validate the use of fMRI imaging biomarkers. The goal of the QIBA fMRI committee is to develop a profile to guide clinical fMRI acquisition and analysis to improve the reproducibility of single‐subject fMRI activation. The identification of data processing methods that improve the reproducibility of specific fMRI activation metrics will help guide the development of this profile.

## 2 MATERIALS AND METHODS

### 2.1 Data

A publicly available test–retest fMRI data set (Gorgolewski, Storkey, Bastin, Whittle, Wardlaw, et al., 2013) was downloaded from the OpenNeuro website (OpenNeuro, 2017) and used for analysis. Each of ten normal, healthy subjects (median age 52.5 years, age range 50–58, four males, six females) was scanned during two sessions, either 2 or 3 days apart. The study was approved by South East Scotland Research Ethics Committee 01.

Subjects performed five behavioral tasks: (1) overt word repetition, (2) covert verb generation, (3) overt verb generation, (4) motor movements for (a) finger, (b) foot, or (c) lips, and (5) (a) a visual landmark identification task or (b) a visual detection task. These tasks are well established through group studies and have potential use for presurgical cortical mapping (Gorgolewski, Storkey, Bastin, Whittle, Wardlaw, et al., 2013). Task timing was understood as follows. The overt word repetition task included four repetition times (TRs) of rest followed by six cycles of six TRs of task and six TRs of rest (76 image volumes total), using sparse sampling for an effective TR of 5 s. The covert verb generation task included five TRs of rest followed by seven cycles of 12 TRs of task and 12 TRs of rest (173 image volumes total). The overt verb generation task included four TRs of rest followed by seven cycles of six TRs of task and six TRs of rest (88 image volumes total), using sparse sampling for an effective TR of 5 s. The motor task included four TRs of rest followed by ten cycles of six TRs of a finger‐tapping task and six TRs of a foot‐tapping task and six TRs of a lip‐moving task (184 image volumes total). The visual task included 10 TRs of rest followed by eight cycles of six TRs of a visual landmark identification task and seven TRs of rest and six TRs of a visual detection task and seven TRs of rest, all of which was followed by 20 TRs of rest (238 image volumes total).

Images were acquired on a GE Signa HDxt 1.5 T scanner with an 8‐channel phased array coil at the Brain Imaging Centre at the University of Edinburgh, UK. All fMRI acquisitions used single‐shot gradient‐echo echo planar images (EPI) with a field of view (FOV) of 256 × 256 mm^2^, slice thickness = 4 mm, 30 slices per image volume, interleaved acquisition order, matrix of 64 × 64, TR = 2.5 s, echo time (TE) = 50 ms, and flip angle = 90°. Thus, the EPI voxel size was 4 × 4 × 4 mm^3^. High‐resolution 3D *T*_1_‐weighted image volumes were acquired in the coronal plane with a FOV = 256 × 256 mm^2^, slice thickness = 1.3 mm, 156 slices, acquisition matrix of 256 × 256, TR = 10s, TE = 4 ms, and inversion time (TI) of 500 ms.

### 2.2 Data analysis

Image data were processed using locally written shell scripts, AFNI (version AFNI_18.0.05) (Cox, 1996), FSL (version 5.0.10) (Smith et al., 2004) and locally written programs written for MATLAB (version R2018b) (The MathWorks, Inc., Natick, MA).

During preprocessing, high‐resolution *T*_1_ image volumes were resampled to 1 × 1×1 mm^3^ resolution (using the AFNI program *3dresample*), deobliqued (using the AFNI program *3dWarp*), and had skull intensities removed (using the AFNI program *3dSkullStrip*). Retest session *T*_1_ image volumes were then aligned to the test session *T*_1_ image volumes (using the AFNI program *3dAllineate*). The *T*_1_ image volumes were then segmented into gray matter, white matter, and cerebrospinal fluid (using the FSL program *fast*) (Zhang, Brady, & Smith, 2001). They were also warped to the MNI152 atlas brain (using the AFNI program *@auto_tlrc* ). The EPI image volumes were deobliqued (using the AFNI program *3drefit*), aligned to the corresponding *T*_1_ image volume (using the AFNI command *align_epi_anat.py*), and had zero‐valued slices removed (using the AFNI command *3dZcutup*).

### 2.3 Motion correction

Four different methods were applied for the stage of motion correction to create four separate streams of data processing, all performed using AFNI (Table 1). The first method was no motion correction (NoMoCo). The second method (2D + 3D) consisted of a 2D slicewise motion correction using the first image as a base image (using the AFNI command *2dImReg*), followed by a 3D rigid motion correction using the first image volume as a base image volume (using the AFNI command *3dvolreg*). The third method (3D Rigid) was a 3D rigid motion correction using the first image volume as the base image volume (using the AFNI command *3dvolreg*). The fourth method (Affine) was an affine motion correction that warped each image volume to the base image volume (using the AFNI command *align_epi_anat.py*). This final motion correction method used a weighted local Pearson coefficient to align and warp each $T_{2}^{*}$‐weighted image volume to a *T*_1_‐weighted image volume (Saad et al., 2009). After motion correction, the initial image volume was removed from each run.

**Table 1:** Data processing methods used

Motion Correction	NoMoCo	2D + 3D	3D Rigid	Affine	
Spatial Smoothing	Blur00	Blur03	Blur06	Blur09	Blur12
Regression	Std	REML	Cen2	Cen5	MPC
Thresholding	Bon	FDR	Clst	AMPLE	

### 2.4 Spatial smoothing

Five different methods were applied for the stage of spatial smoothing to create five new streams of data processing for each preceding stream (Table 1). The first method was no spatial smoothing (Blur00). The next four methods (Blur03, Blur06, Blur09, and Blur12) included four different spatial smoothing kernels (full width at half maximum (FWHM) of 3, 6, 9, and 12 mm) (using the AFNI command *3dBlurInMask*), creating four new sets of data. Spatial smoothing was only applied within a brain mask made for each subject's run.

### 2.5 Regression

Five different methods were applied for the stage of regression to create five new streams of data processing for each preceding stream (Table 1). Regression was performed using a brain mask, a stimulus time series (the stimulus function convolved with a hemodynamic response function), and a baseline polynomial (of order 1 for <150 volumes and order 2 for equal to or greater than 150 volumes) (using the AFNI command *3dDeconvolve*). Five different variations were employed to reflect common regression methods found in the literature. The first method (Std) was a standard regression using the aforementioned options. The second method (REML) used a restricted maximum likelihood (REML) estimation of the temporal autocorrelation structure to account for temporal autocorrelations (using the AFNI command *3dREML*). The third method (Cen2) censored time points that had a framewise displacement (FD) greater than 0.2 (a strict threshold). The fourth method (Cen5) censored time points that had an FD greater than 0.5 (a lenient threshold). The fifth method (MPC) used all six motion parameters from the 3D rigid motion correction method as covariates (i.e., nuisance regressors).

### 2.6 Threshold

**Figure 1:**
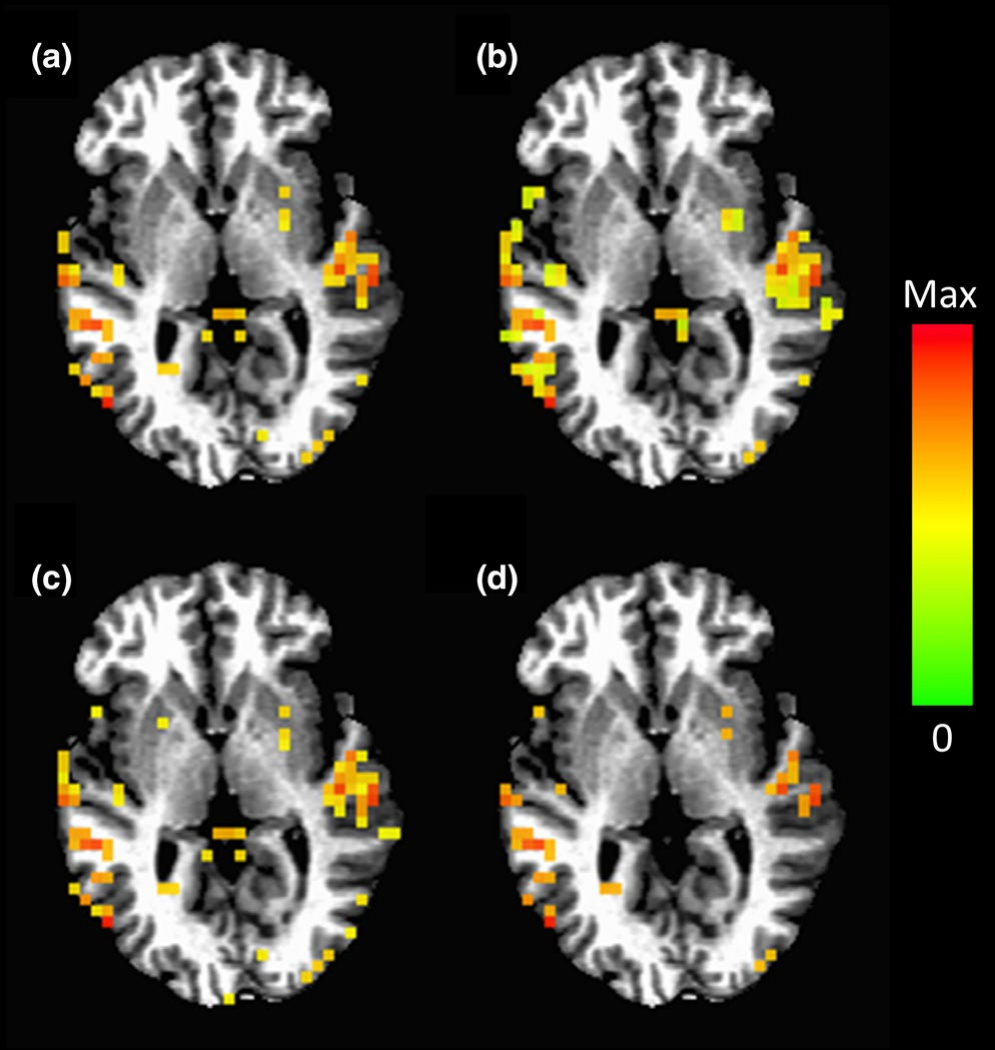
Example activation maps for subject 1, session 1, and run 1 showing comparable activation using (a) a Bonferroni threshold of *p*_c_ < .01, (b) a cluster threshold of *p*_c_ < .01, (c) an FDR threshold of *p*_FDR_ < 1 × 10^–5^, and (d) an AMPLE threshold of AMPLE > 60%

Four different methods were applied for the stage of thresholding to create four new streams of data processing for each preceding stream (Table 1). The first method (Bon) was the Bonferroni threshold (a familywise error rate), where the *p*‐value was multiplied by the total number of voxels in the mask data set, and activation maps were achieved using a corrected threshold of *p*_c_ < .01. The second method (Clst) was a cluster threshold. In this method, the temporal autocorrelation function parameters were first determined (using the AFNI command *3dFWHMx*). Next, these values, the brain mask, and a voxelwise threshold of *p* < .0001 were used to determine cluster size volumes (using the AFNI command *3dClustSim*), which were then applied to the activation map (using the AFNI command *3dclust*), and activation maps were achieved using a corrected threshold of *p*_c_ < .01. The third method (FDR) was the false discovery rate (FDR) threshold method (Genovese, Lazar, & Nichols, 2002), where activation maps were achieved using a corrected threshold of *p*_FDR_ < 1 × 10^–5^. (A more stringent threshold was used for FDR, as it represents a much more sensitive thresholding technique.) The fourth method (AMPLE) was the amplitude mapping as a percentage of local excitation (AMPLE) thresholding method (Voyvodic, 2012; Voyvodic, Petrella, & Friedman, 2009). In this method, the brain was divided into five separate regions (frontal, occipital, parietal, temporal, and other), and the maximum *t*‐statistic was found in each region. In each region, all of the *t*‐statistics were divided by the maximum *t*‐statistic of that region and multiplied by 100% to yield AMPLE values. Activation maps were then achieved using a threshold of AMPLE > 60%. Figure 1 shows comparable activation maps for subject 1, session 1, and run 1 using the four different thresholding methods.

### 2.7 Cluster identification and matching

Combining all the possible combinations of analyses resulted in 400 analyses for each activation map. Because there were five runs, one with three activations and one with two activations, this resulted in a total of 400 analyses × 8 activation maps × 10 subjects × 2 sessions = 64,000 activation maps. With this many activation maps, it would be impractical to identify activation clusters manually. Therefore, a program was written to automatically identify activation clusters.

First, region of interest (ROI) masks were created. Each run's activation map was warped to the MNI152 atlas brain using warping parameters used to warp the *T*_1_ image volume to the atlas brain. For each type of activation, the activation maps were combined across 400 analyses, 10 subjects, and two sessions in the atlas space. Voxels that were active in at least 10% of the runs were then included in the ROI mask. The ROI mask was then warped to subject space for each subject.

Next, a program was employed to automatically identify activation clusters using the ROI masks. For each activation map, the routine identified all activation clusters with a minimum cluster size of 12 voxels (768 μl) with a weighted center of mass (CoM) inside the ROI mask. This was repeated for all 400 analyses, both sessions, and all ten subjects (or 8,000 activation maps).

After the clusters were identified, another program was used to match clusters from the test session to clusters in the retest session. Essentially, for each cluster in the test run, the routine identified the closest cluster in the retest run that had a CoM less than or equal to 10 mm from the CoM in the test run.

### 2.8 Test–retest metrics

Three test–retest metrics were calculated. These metrics were calculated using the activation cluster pairs identified with methods presented above. The first test–retest metric was the percent difference in activation volume (PDAV):

$$\text{PDAV}=\frac{|V_{1}-V_{2}|V_{1}+V_{2}/2}{\times }100\%,$$

using the volume from the cluster in session 1 (*V*_1_) and the volume from the cluster in session 2 (*V*_2_). Ideally, the percent difference in activation volume would be 0%.

The second test–retest metric was the difference in the center of mass (DCM) between test and retest runs. This was calculated as the Euclidean distance between the two centers of mass, weighted by the *t*‐statistics, given by coordinates (*x*_1_, *y*_1_, *z*_1_) and (*x*_2_, *y*_2_, *z*_2_):

$$\text{DCM}=\sqrt{{\left(x_{1}-x_{2}\right)}^{2}+{\left(y_{1}-y_{2}\right)}^{2}+{\left(z_{1}-z_{2}\right)}^{2}}.$$

Ideally, the difference in the center of mass would be 0 mm.

The third test–retest metric was the Dice Similarity Coefficient (DSC), which measures the degree of overlap between two regions, *C*_1_ and *C*_2_ (Dice, 1945):

$$\text{DSC}=\frac{2\times (C_{1}\cap C_{2})}{C_{1}+C_{2}}$$

This can be understood as two times the number of voxels in the intersection of the two clusters divided by the total number of voxels in both clusters. Ideally, the DSC would be 1.

All three test–retest metrics were computed for all the identified clusters pairs. Next, distributions and means of the test–retest metrics were computed. The distributions and means were compared across the four different motion correction methods, the five different spatial smoothing methods, the five different regression methods, and the four different thresholding methods. Kruskal–Wallis tests were performed to see whether the distributions were different across different processing methods. Next, unpaired *t* tests were performed to see whether the mean test–retest metrics, computed across subjects and all activation maps, were significantly different across processing methods (accounting for multiple comparisons across the number of processing methods and activation maps). Box plots were also created to visualize the distribution of the test–retest metrics across methods. Lastly, effect sizes were calculated using Cohen's *d*, where *d* equals the mean difference divided by the pooled standard deviation:

$$d=\frac{\bar{x_{1}}-\bar{x_{2}}}{\sqrt{\left(\left(n_{1}-1\right)s_{1}^{2}+\left(n_{1}-1\right)s_{2}^{2}\right)/\left(n_{1}+n_{2}\right)}}$$

## 3 RESULTS

**Figure 2:**
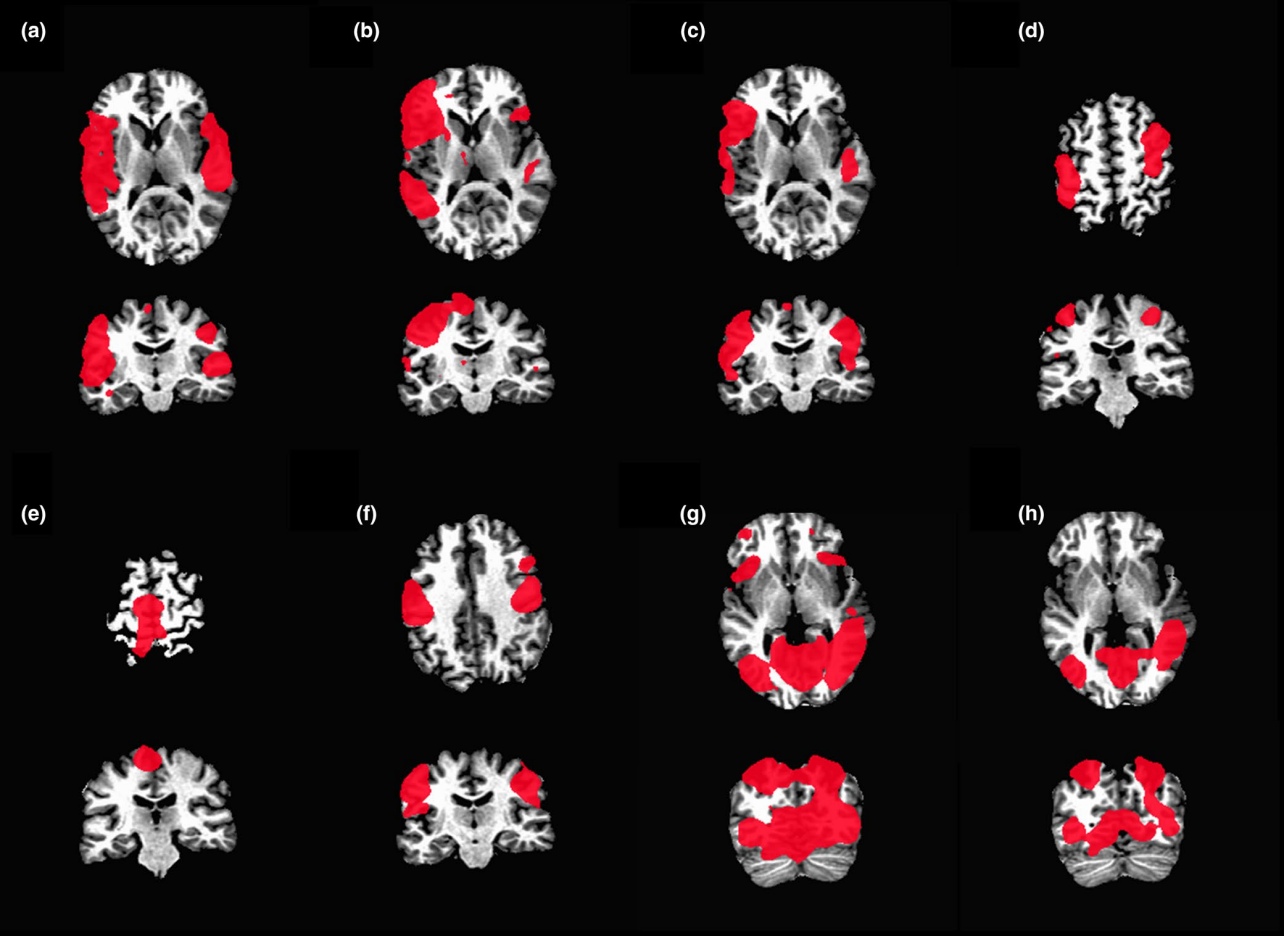
ROI masks for (a) run 1, overt word repetition, (b) run 2, covert verb generation, (c) run 3, overt verb generation, (d) run 4a, finger‐tapping, (e) run 4b, foot‐tapping, (f) run 4c, lip‐moving, (g) run 5a, visual landmark identification task, and (h) run 5b, visual detection task

Figure 2 shows examples of the ROI masks for all activation maps warped to subject 1 space. It can be seen that the ROIs for the language tasks (Figure 2a–c) contain regions in Broca's area and Wernicke's area, as expected. The ROI mask for the finger‐tapping task includes the medial precentral gyrus (Figure 2d). The ROI mask for the foot‐tapping task includes the superior precentral gyrus (Figure 2e). The ROI mask for the lip‐moving task includes the inferior precentral gyrus (Figure 2f). And the ROI masks for the visual landmark identification and detection tasks include the visual cortex and parietal lobes (Figure 2g,h).

Results of the Kruskal–Wallis tests, shown in Tables 2, 3, 4, 5, reveal that, in most cases, the distributions of the test–retest metrics were different across the methods. These results supported the decision to run unpaired *t* tests comparing the mean test–retest metric from one method to another.

**Table 2:** Results of the Kruskal–Wallis tests determining if the distributions of the test–retest metrics were significantly different across different motion correction methods

	Run	PDAV	DCM	DSC
Language	Run 1	***p* = 3.76 × 10^–5^**	***p* = 2.02 × 10^–14^**	***p* = 7.78 × 10^–31^**
	Run 2	***p* = 2.14 × 10^–2^**	***p* = 9.13 × 10^–23^**	***p* = 3.40 × 10^–32^**
	Run 3	***p* = 2.60 × 10^–2^**	***p* = 2.22 × 10^–3^**	***p* = 3.63 × 10^–10^**
Motor	Run 4a	*p* = 2.33 × 10^–1^	***p* = 4.08 × 10^–7^**	***p* = 2.91 × 10^–23^**
	Run 4b	*p* = 1.43 × 10^–1^	***p* = 1.94 × 10^–8^**	***p* = 1.64 × 10^–11^**
	Run 4c	***p* = 2.38 × 10^–5^**	***p* = 3.03 × 10^–11^**	***p* = 3.44 × 10^–23^**
Visual	Run 5a	*p* = 9.20 × 10^–1^	***p* = 1.31 × 10^–11^**	***p* = 3.72 × 10^–38^**
	Run 5b	*p* = 4.78 × 10^–1^	***p* = 6.03 × 10^–15^**	***p* = 8.26 × 10^–43^**

Note

Bold values indicate a significance of *p* < .05.

**Table 3:** Results of the Kruskal–Wallis tests determining if the distributions of the test–retest metrics were significantly different across different spatial smoothing methods

	Run	PDAV	DCM	DSC
Language	Run 1	3.67 × 10^–1^	**3.84 × 10^–9^**	**3.39 × 10^–10^**
	Run 2	1.42 × 10^–1^	3.54 × 10^–1^	**7.42 × 10^–20^**
	Run 3	5.62 × 10^–2^	**2.14 × 10^–2^**	**1.13 × 10^–6^**
Motor	Run 4a	**7.83 × 10^–29^**	**3.17 × 10^–25^**	2.07 × 10^–1^
	Run 4b	**8.37 × 10^–10^**	**2.83 × 10^–15^**	5.60 × 10^–1^
	Run 4c	**4.79 × 10^–28^**	**2.42 × 10^–7^**	**1.72 × 10^–3^**
Visual	Run 5a	**4.29 × 10^–8^**	**2.25 × 10^–8^**	**9.38 × 10^–8^**
	Run 5b	**3.18 × 10^–13^**	6.13 × 10^–1^	**5.41 × 10^–11^**

Note

Bold values indicate a significance of *p* < .05.

**Table 4:** Results of the Kruskal–Wallis tests determining if the distributions of the test–retest metrics were significantly different across different regression methods

	Run	PDAV	DCM	DSC
Language	Run 1	2.09 × 10^–1^	1.57 × 10^–1^	**1.01 × 10^–2^**
	Run 2	**1.47 × 10^–10^**	9.24 × 10^–2^	**2.37 × 10^–8^**
	Run 3	**1.88 × 10^–4^**	1.96 × 10^–1^	**1.74 × 10^–6^**
Motor	Run 4a	**1.31 × 10^–3^**	**1.84 × 10^–4^**	**4.69 × 10^–2^**
	Run 4b	**6.22 × 10^–6^**	**6.31 × 10^–8^**	**1.27 × 10^–6^**
	Run 4c	**7.98 × 10^–6^**	**1.20 × 10^–5^**	**2.94 × 10^–11^**
Visual	Run 5a	**1.71 × 10^–9^**	**4.71 × 10^–2^**	**9.16 × 10^–9^**
	Run 5b	**1.35 × 10^–3^**	4.98 × 10^–1^	**2.11 × 10^–2^**

Note

Bold values indicate a significance of *p* < .05.

**Table 5:** Results of the Kruskal–Wallis tests determining if the distributions of the test–retest metrics were significantly different across different thresholding methods

	Run	PDAV	DCM	DSC
Language	Run 1	**1.84 × 10^–3^**	**6.60 × 10^–7^**	4.33 × 10^–1^
	Run 2	**7.07 × 10^–5^**	**3.64 × 10^–4^**	4.08 × 10^–1^
	Run 3	**1.06 × 10^–7^**	**1.11 × 10^–6^**	**6.88 × 10^–3^**
Motor	Run 4a	**1.51 × 10^–7^**	7.86 × 10^–1^	6.53 × 10^–1^
	Run 4b	**6.62 × 10^–12^**	**9.24 × 10^–3^**	**8.90 × 10^–7^**
	Run 4c	**8.45 × 10^–11^**	**6.52 × 10^–3^**	**3.30 × 10^–6^**
Visual	Run 5a	**4.15 × 10^–8^**	3.29 × 10^–1^	8.30 × 10^–1^
	Run 5b	7.24 × 10^–2^	5.60 × 10^–2^	**2.16 × 10^–2^**

Note

Bold values indicate a significance of *p* < .05.

**Figure 3:**
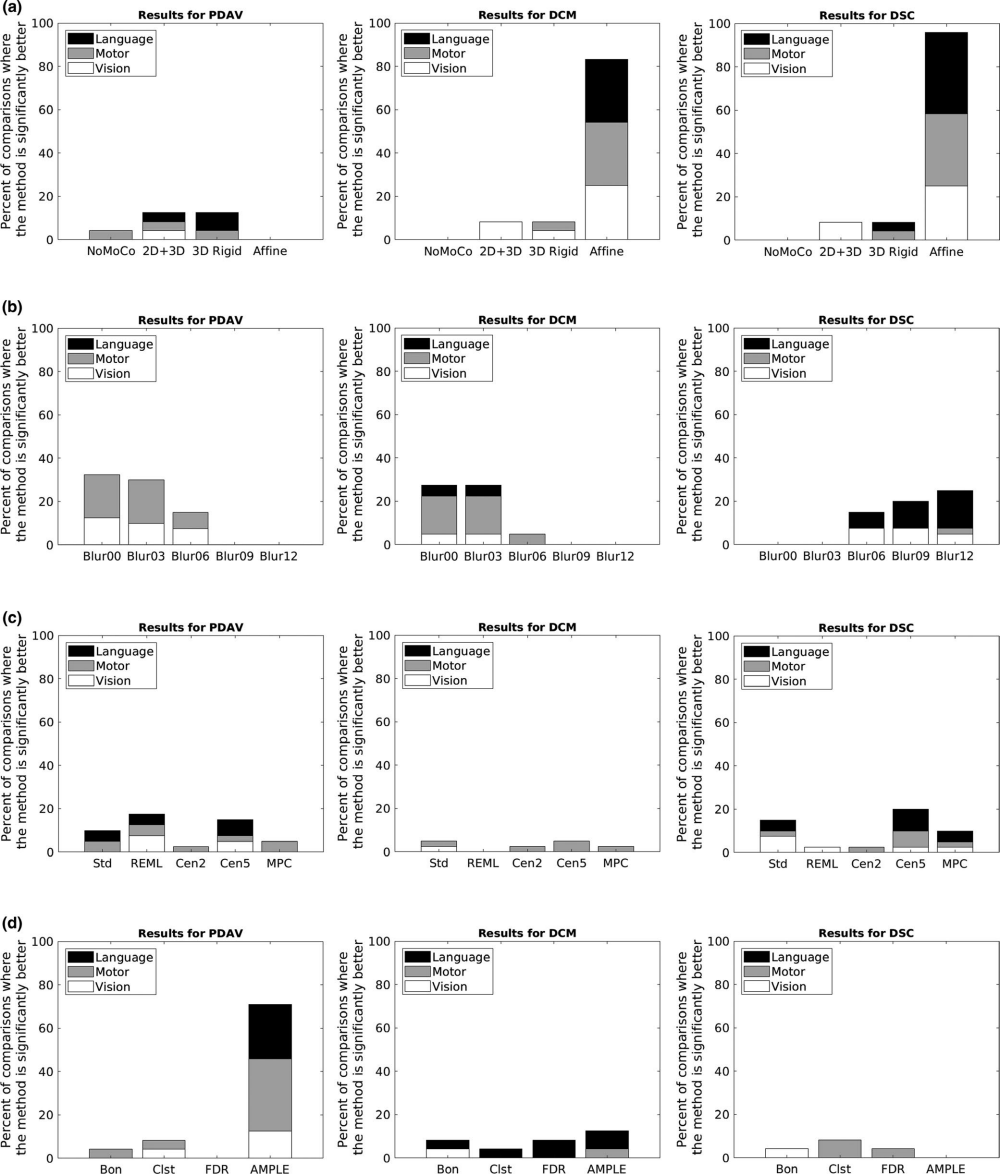
Plots for the percent of comparisons where the method was significantly better for (left column) the percent difference in activation volume (PDAV), (middle column) the difference in center of mass (DCM), and (right column) the Dice Similarity Coefficient (DSC) for different (a) motion correction methods, (b) spatial smoothing methods, (c) regression methods, and (d) thresholding methods

**Figure 4:**
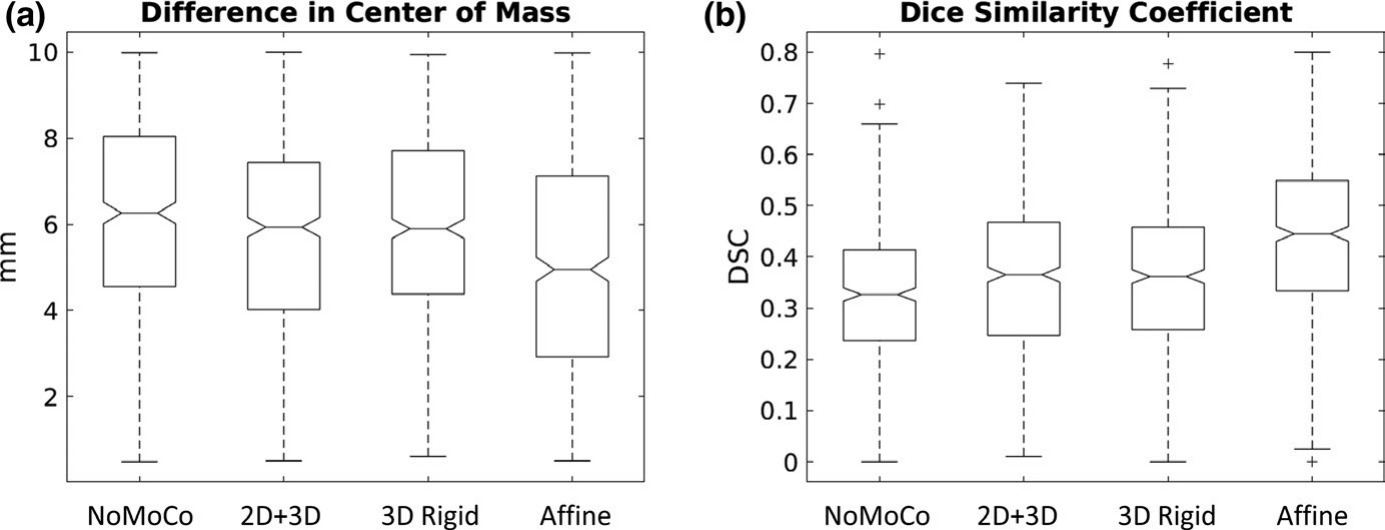
Example box plots across different motion correction methods for (a) the difference in center of mass for run 1 (overt word repetition) and (b) the Dice Similarity Coefficient for run 1 (overt word repetition)

**Figure 5:**
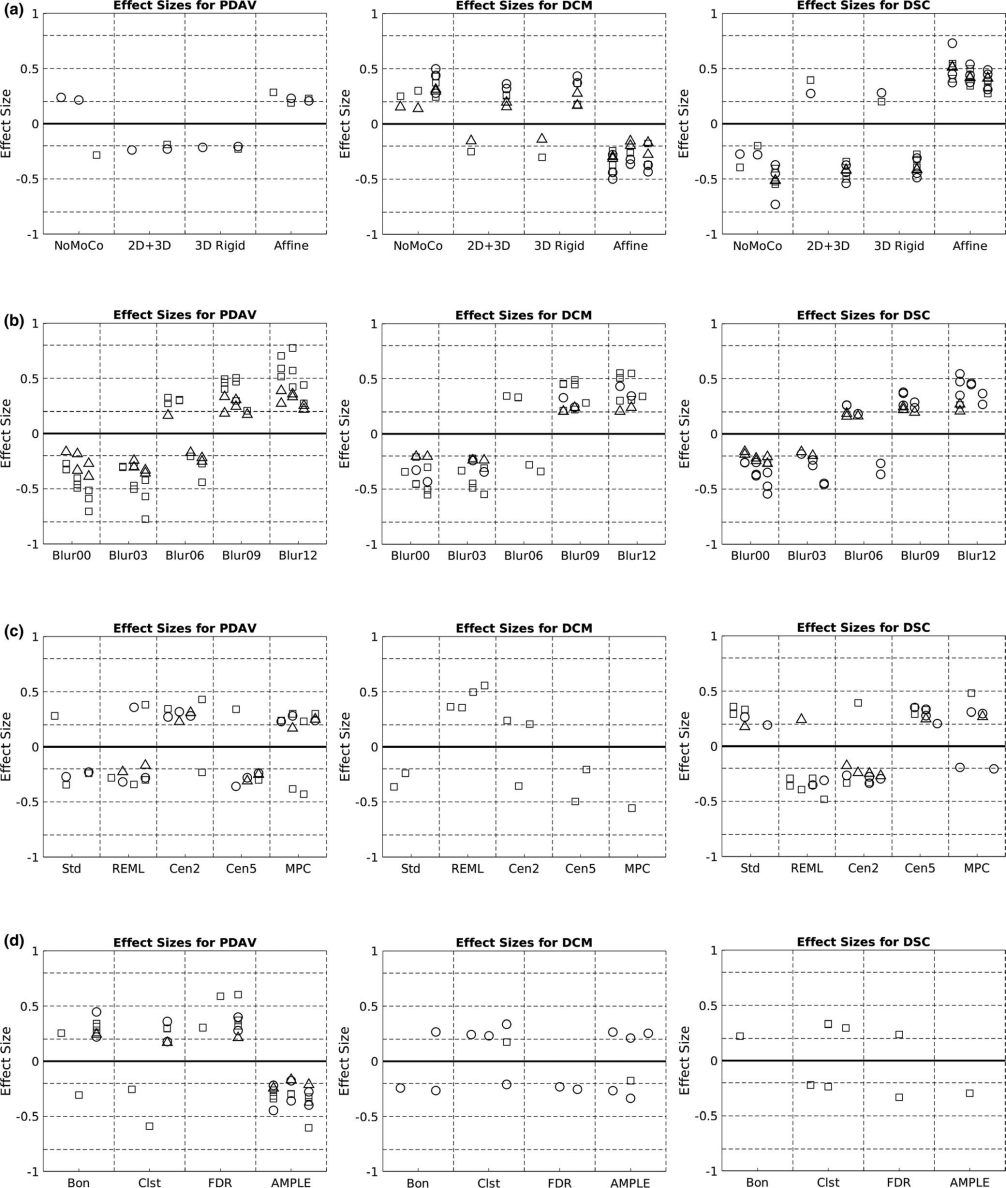
Plots of effect size for (left column) the percent difference in activation volume (PDAV), (middle column) the difference in center of mass (DCM), and (right column) the Dice Similarity Coefficient (DSC) across (a) motion correction methods, (b) spatial smoothing methods, (c) regression methods, and (d) thresholding methods. Only significantly different (*p* < .05) cases are shown. Circles indicate language task, squares indicate motor task, and triangles indicate vision task. Horizontal dashed lines indicate ranges of effect size, from very small (0–0.2), small (0.2–0.5), medium (0.5–0.8), and large (0.8 and up)

For the comparisons across different motion correction methods, three methods yielded mean values for PDAV that were significantly better (*p* < .05) than all the other methods (Figure 3a [left]). However, only a small percentage of cases were significantly better, and no method stood out as being better than the others. Figure 3a (middle) shows that affine motion correction yielded a significantly better (*p* < .05) DCM for 83% of all possible comparisons, with cases distributed evenly across language, motor, and vision tasks. As an example, a box plot for the difference in center of mass across the four motion correction methods for run 1 is shown in Figure 4a. For this case, the median DCM for affine motion correction was clearly lower than that for the other three methods, especially no motion correction. Figure 3a (right) shows that affine motion correction yielded a significantly better (*p* < .05) DSC for 96% of the comparisons, with cases distributed evenly across language, motor, and vision tasks. 2D + 3D and 3D rigid motion correction also yielded significantly better DSC values, but for smaller numbers of comparisons. As an example, a box plot for the DSC across the four motion correction methods for run 1 is shown in Figure 4b. For this case, the median DSC for affine motion correction was clearly greater than that for the other three methods, especially no motion correction. The effect sizes for all of the significant comparisons between motion correction methods are plotted in Figure 5a. A positive value indicates that column's method yielded a test–retest metric that was significantly greater than the other method. A negative value indicates that that column's method yielded a test–retest metric that was significantly less than the other method. Figure 5a (middle) shows that affine motion correction consistently yielded a DCM that was significantly smaller than other methods with effect size magnitudes ranging from very small to small. Likewise, Figure 5a (right) shows that affine motion correction consistently yielded DSC values that were significantly greater than other methods with effect size magnitudes ranging from small to medium.

**Figure 6:**
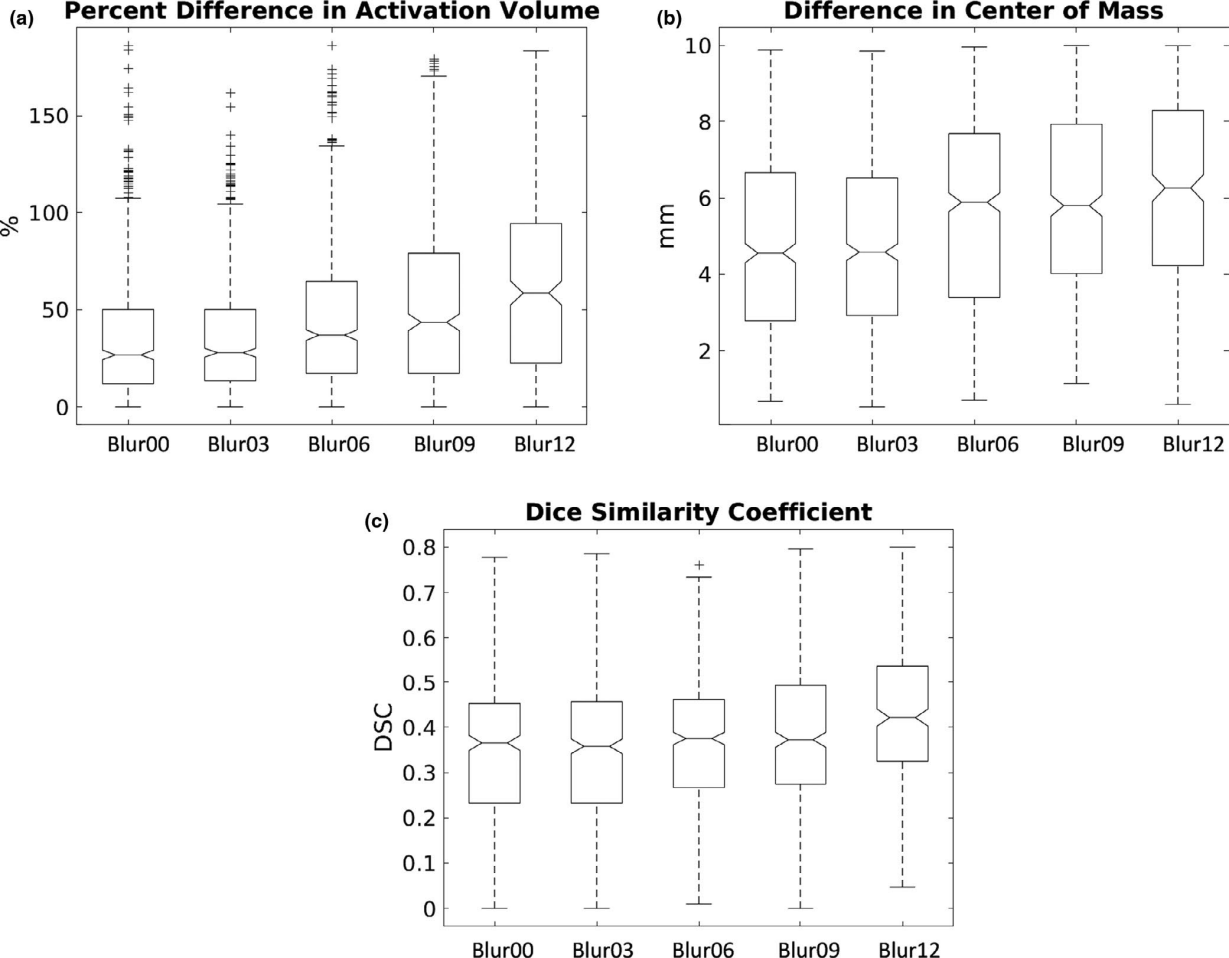
Example box plots across different spatial smoothing methods for (a) the percent difference in activation volume for run 4a (finger‐tapping), (b) the difference in center of mass for run 4a (finger‐tapping), and (c) the Dice Similarity Coefficient for run 1 (overt word repetition)

For the comparisons across different spatial smoothing methods, there was a trend toward methods with less smoothing yielding more comparisons where the mean value for the PDAV was significantly better (*p* < .05) (Figure 3b [left]). Oddly, no clusters from language contributed to this result. As an example, a box plot of the PDAV across the five spatial smoothing methods for run 4a (finger‐tapping) is shown in Figure 6a. There was a clear trend toward decreasing values of PDAV as spatial smoothing decreased. Similarly, Figure 3b (middle) shows that no smoothing (Blur00) or smoothing with a 3 mm FWHM (Blur03) both yielded 28% of the comparisons where the mean value for DCM was significantly better (*p* < .05). As an example, a box plot of the DCM across the five spatial smoothing methods for run 4a (finger‐tapping) is shown in Figure 6b. There was a clear trend toward decreasing values of DCM as spatial smoothing decreased. In contrast, Figure 3b (right) shows that the three methods with the most spatial smoothing (FWHM of 6, 9, and 12 mm) each had between 15% and 25% of the comparisons of the DSC being significantly better (*p* < .05). As an example, a box plot of the DSC values across the five spatial smoothing methods for run 1 is shown in Figure 6c. There was a clear trend toward increasing values of DSC as spatial smoothing increased. The effect sizes for all of the significant comparisons between spatial smoothing methods are plotted in Figure 5b. For all three test–retest metrics, there was a trend toward less spatial smoothing yielding smaller values for each test–retest metric. The cases of Blur00 and Blur03 both yielded negative effect sizes for all significant comparisons with other smoothing sizes. Thus, Blur00 and Blur03 both yielded PDAV, DCM, and DSC values that were lower than other methods for all comparisons that were significantly different.

For the comparisons across different regression methods, all five methods had at least one case where the mean values for PDAV were significantly better (*p* < .05) than other methods (Figure 3c [left]). However, only a small percentage of cases were significantly better, and no method stood out as being better than the others. Figure 3c (middle) shows that four methods yielded mean values for DCM that were significantly better (*p* < .05) than other methods. However, only a small percentage of cases were significantly better, and no method stood out as being better than the others. Figure 3c (right) shows that all five regression methods had at least one case where DSC was significantly better (*p* < .05) than other methods. However, no method stood out as being better than the others. The effect sizes for all significant comparisons between regression methods are plotted in Figure 5c. For all three test–retest metrics, the effect size magnitude generally ranged from very small to small.

**Figure 7:**
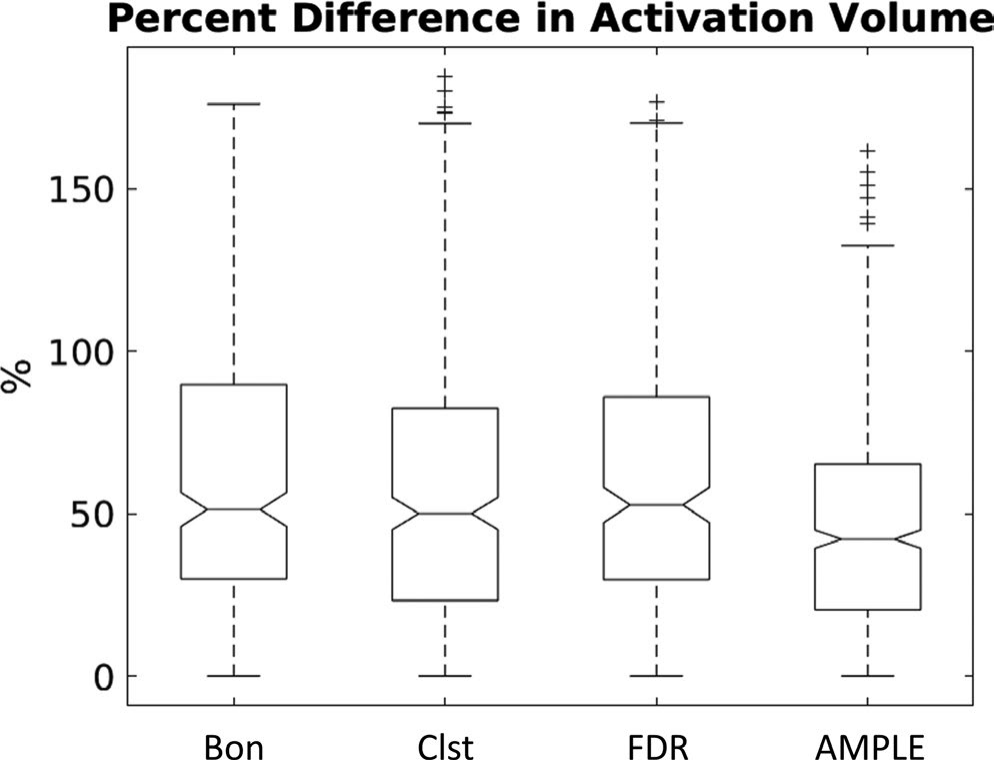
Example box plot across different thresholding methods for the percent difference in activation volume for run 3 (overt verb generation)

For the comparisons across different thresholding methods, AMPLE thresholding yielded a mean value for PDAV was significantly better (*p* < .05) than other methods in 71% of comparisons (Figure 3d [left]). Figure 7 shows an example box plot of the PDAV across the four thresholding methods for run 3 (overt verb generation). For this case, the median PDAV was clearly lower for AMPLE thresholding compared to the other thresholding methods. Figure 3d (middle) shows that all four methods had cases where the mean DCM was significantly better (*p* < .05) than other methods. However, only a small percentage of cases were significantly better, and no method stood out as being predominantly better than the others. Figure 3d (right) shows that three methods had cases where the mean DSC was significantly better (*p* < .05) than other methods. However, only a small percentage of cases were significantly better, and no method stood out as being predominantly better than the others. The effect sizes for all significant comparisons between thresholding methods are plotted in Figure 5d. Figure 5d (left) shows that AMPLE thresholding consistently yielded PDAV values that were significantly less than other methods with effect size magnitudes ranging from very small to medium. For the other test–retest metrics, the effect size magnitude generally ranged from small to medium.

## 4 DISCUSSION

In this study, a publicly available test–retest fMRI data set was used to examine the effect of different data processing steps on the reproducibility of single‐subject fMRI activation maps. Comparisons were made across four different motion correction methods, five different spatial smoothing methods, five different regression methods, and four different thresholding methods. This study was not meant to be a comprehensive examination of all possible methods, but rather a comparison study of popular methods found in the literature. The goal was to see whether, given a limited set of data processing methods, specific ones could improve the reproducibility of fMRI activation when compared to other methods. Future studies can be used to compare other data processing methods and different statistical thresholds.

One significant finding was that affine motion correction yielded more reproducible results for the weighted center of mass. This was true for 83% of the comparisons between affine motion correction and all other motion correction methods across all eight activation maps. Significant results were found across language, motor, and vision tasks. The effect sizes of these comparisons ranged from very small to small. This means that, even though affine motion correction yielded significantly better values for the difference in the weighted center of mass, the differences might not always be noticeable. Similarly, affine motion correction yielded better results for the Dice Similarity Coefficient. This was true for 96% of the comparisons between affine motion correction and all other motion correction methods across all eight activation maps. For this test–retest metric as well, significant results were found across language, motor, and vision tasks. The effect sizes of these comparisons ranged from very small to medium. Even though affine motion correction yielded significantly better DSC values, the improvement might not always be noticeable. Because motion can occur within the acquisition of a single head volume, resulting EPI head volumes may end up spatially distorted. Therefore, it is reasonable to expect that affine motion correction, which can scale and shear individual image slices, would yield an advantage over standard, rigid head motion correction methods for improving the reproducibility of spatial information of fMRI activation. Despite the small effect sizes calculated, affine motion correction is likely to account for intravolume distortions and improve the reproducibility of the weighted center of mass and the overlap of fMRI activation clusters.

Another significant finding is that less spatial smoothing yielded more reproducible results for both the activation volume and the weighted center of mass. Spatial smoothing may be commonly used because of its ability to make “cleaner‐looking” activation maps. One survey reported that 81% of sites performing fMRI for epilepsy surgical planning use spatial smoothing, most commonly employing a FWHM of 8 mm (Benjamin et al., 2018). Nevertheless, the current study confirmed findings from previous studies that showed how spatial smoothing can cause a loss of fMRI spatial information (Fransson et al., 2002; Geissler et al., 2005). Interestingly, the complete lack of spatial smoothing did not dominate for improving reproducibility. Rather, smoothing kernels of decreasing size showed increasing numbers of cases of increased reproducibility. Therefore, to improve the reproducibility of activation volume and the weighted center of mass, it may be best to use either no spatial smoothing or very little (e.g., FWHM of 3 mm) in fMRI analysis. This finding is complementary to another reason to avoid spatial smoothing in that it can affect gyral localization, which is important to presurgical planning.

Conversely, increased spatial smoothing was found to increase the Dice Similarity Coefficient, thus increasing the overlap of activation volumes between sessions. Excessive spatial smoothing can actually be problematic, however, as it leads to a blurring of the activation that may encompass the entire local gyral space. Because the activation clusters were bounded by a brain mask, increased spatial smoothing will naturally lead to high overlap values (and a concurrent decrease in specificity), but this will be due to an artifact of the analysis method rather than an optimization. This reasoning casts doubt on the utility of the overlap coefficient as a suitable index for examining fMRI activation reproducibility. In fact, this reasoning may explain why lower thresholding improved the overlap index in previous reliability studies (Duncan et al., 2009; Fernandez et al., 2003; Nettekoven et al., 2018; Stevens et al., 2013).

Although statistical tests showed differences in the distributions of the test–retest metrics for different regression methods, there was no regression method that stood out as more likely to improve the reproducibility of fMRI activation. Figure 3c (right) hinted that standard regression or regression with censoring time points with FD > 0.5 might sometimes yield a better DSC. However, these results were only true for small fractions of the comparisons. Therefore, based on the regression methods tested, it is unclear if there is a regression method that can improve the reproducibility of fMRI activation. It is possible that downstream methods like regression may be subject to so much variability that they offer less ability for optimization when it comes to reproducibility.

Another major finding of the current study was that AMPLE thresholding yielded more reproducible results for the activation volume than the other thresholding methods. This was true for 71% of the comparisons between AMPLE thresholding and all other methods. This result confirms findings from previous studies focusing on motor (Voyvodic et al., 2009) and language (Voyvodic, 2012) activation. However, no thresholding method was found to improve the reproducibility of the weighted center of mass or the overlap.

A previous study has shown that increasing the overall threshold can increase reproducibility of the center of mass (Fesl et al., 2008). However, the current study focused on fixed thresholding methods instead of varying the threshold stringency. It is believed that reasonable fixed thresholds offer the best balance between sensitivity and specificity. For the application of presurgical planning, increasing the threshold stringency runs the risk of lowering the sensitivity of the analysis, which could result in harm to patients if undetected eloquent cortex is removed.

There is an assumption that there exists a single data analysis pipeline that can optimize the reproducibility of fMRI activation. The current study contradicted this assumption by revealing that the methods needed to improve the reproducibility of the fMRI activation depend on the fMRI activation metric of interest. To improve the reproducibility of activation volume, it was found optimum to use minimal spatial smoothing and AMPLE thresholding. To improve the reproducibility of the weighted center of mass, it was found optimum to use affine motion correction and minimal spatial smoothing. And to improve the reproducibility of the overlap, it was found optimum to use affine motion correction and maximal spatial smoothing. It was also determined that, due to the potential for excessive spatial smoothing to create activation clusters that fill up the gyral volume, it may not be wise to use overlap as a reproducibility metric. Nevertheless, the data processing methods needed to improve reproducibility were found to depend on the fMRI activation metric of interest.

One issue with the results is that the effect size of the change in the test–retest metric generally ranged from small to medium (Figure 5). This finding suggests that employing the optimum method might not necessarily improve the reproducibility of activation for a given fMRI run. However, even with the range of effect sizes discovered, if you scan a large number of subjects, there will eventually be subjects for which the effect size of the improvement in reproducibility will be large enough to be seen. Furthermore, improvement of reproducibility can be performed by optimizing many different variables. Bennett and Miller (2010, 2013) argued that fMRI reproducibility could be improved by using a well‐maintained scanner, well‐designed tasks, training subjects in a mock scanner, and taking advantage of longer scan time when possible. It is unknown what the effect size each of these factors on its own would have on increasing the reproducibility of fMRI activation metrics. However, when applied together, a combination of several methods is likely to have a large effect size on the improvement in the reproducibility of single‐subject fMRI activation metrics.

This study relied on several assumptions in order to extract large numbers of activation clusters across many combinations of analysis in order to run statistical inferences about different data analysis methods. If any of these assumptions were incorrect, it would affect the conclusions of the study. One assumption was that activation clusters with a minimum cluster size of 12 voxels (768 μl) with a weighted center of mass inside the ROI mask should be identified as clusters of interest. Another assumption was that activation clusters in the test data set should be matched with activation clusters in the retest data set that have weighted centers of mass located within 10 mm. It is believed that these were reasonable assumptions to make. It is unlikely that slight changes to these values would have affected the results very much. Another issue is that the cluster matching method allowed the possibility that a cluster in one session could be matched with more than one cluster in the other session, which could have affected the analysis. Furthermore, it is possible that some of the identified cluster pairs do not represent identical neural activity centers in the brain. The overall assumption was that, out of the many hundreds of cluster pairs identified, the number of true cluster pairs would have outnumbered the possible false cluster pairs, yielding results approximating the truth.

The current study was also limited by the data set used. The data were acquired on a 1.5 T MR scanner using an 8‐channel phased array coil. It is unclear if the results would apply to data acquired on a 3 T MR scanner where the SNR is higher. Another limitation was the size of the data set used. The publicly available test–retest fMRI data set included data from ten subjects participating in five different task‐based runs. The data from these ten subjects may not necessarily represent all the possible types of motion, physiological noise, and other types of variability present in fMRI data. A larger test–retest data set of task‐based fMRI data would have provided a more comprehensive set of data to study. For example, none of the regression methods stood out as being predominantly better than the others. This could be because, as the strengths and weaknesses of these methods are added up, the different methods ended up being quite similar to one another. Alternatively, it could be that one of the regression methods would have been better at increasing the reproducibility of fMRI activation if the subjects exhibited more motion. Task‐based fMRI data sets with greater numbers of subjects will be needed to explore these issues. However, such a data set is not yet available for analysis.

## 5 CONCLUSIONS

This study identified different data processing methods to improve the reproducibility of three single‐subject fMRI activation metrics. Minimal spatial smoothing and AMPLE thresholding improved the reproducibility of activation volume. Affine motion correction and minimal spatial smoothing improved the reproducibility of the weighted center of mass. Affine motion correction and generous spatial smoothing improved the reproducibility of activation overlap. However, it may not be prudent to use activation overlap as a test–retest metric, as any method that increases sensitivity (and lowers specificity) can artificially increase the overlap.

This study revealed that the data processing methods needed to improve reproducibility depended on the fMRI activation metric of interest. Future studies on fMRI reproducibility should decide a priori on which fMRI activation metrics to study. It would be more beneficial to examine the reproducibility of fMRI activation metrics that have clinical importance rather than examining the reproducibility of voxelwise metrics.

The results of this study can be applied to the use of presurgical fMRI to improve the accuracy of fMRI activation maps and to increase the confidence with which they are used to help neurosurgeons avoid the removal of critical motor and language regions of the brain. The results can also be applied to the RSNA QIBA fMRI effort to create a profile to improve the reproducibility of single‐subject fMRI activation and its application as a quantitative imaging biomarker.

## CONFLICT OF INTEREST

The author declares that there were no conflicts of interest with respect to the authorship or the publication of this article.

## AUTHOR CONTRIBUTIONS

The author (D. Soltysik) analyzed the data, wrote the manuscript, and approved the final version for publication.

## DISCLAIMER

The mention of commercial products, their sources, or their use in connection with material reported herein is not to be construed as either an actual or suggested endorsement of such products by the Department of Health and Human Services.

## Data Availability

Data for this study were downloaded from the OpenNeuro repository at the following link: https://openneuro.org/datasets/ds000114/versions/00001 (accessed 5 Jul 2018). Code will be available upon request.
